# Multivariate Analysis Using High Definition Flow Cytometry Reveals Distinct T Cell Repertoires between the Fetal–Maternal Interface and the Peripheral Blood

**DOI:** 10.3389/fimmu.2014.00033

**Published:** 2014-02-05

**Authors:** Michelle A. Neller, Brigitte Santner-Nanan, Rebekah M. Brennan, Peter Hsu, Steven Joung, Ralph Nanan, Scott R. Burrows, John J. Miles

**Affiliations:** ^1^Human Immunity Laboratory and Cellular Immunology Laboratory, QIMR Berghofer Medical Research Institute, Brisbane, QLD, Australia; ^2^Nepean Centre for Perinatal Care, Sydney Medical School, The University of Sydney, Sydney, NSW, Australia; ^3^School of Medicine, The University of Queensland, Brisbane, QLD, Australia; ^4^Institute of Infection and Immunity, Cardiff University School of Medicine, Cardiff, UK

**Keywords:** T cell repertoire, pregnancy, decidual mononuclear cells, pre-eclampsia

## Abstract

The human T cell compartment is a complex system and while some information is known on repertoire composition and dynamics in the peripheral blood, little is known about repertoire composition at different anatomical sites. Here, we determine the T cell receptor beta variable (TRBV) repertoire at the decidua and compare it with the peripheral blood during normal pregnancy and pre-eclampsia. We found total T cell subset disparity of up to 58% between sites, including large signature TRBV expansions unique to the fetal–maternal interface. Defining the functional nature and specificity of compartment-specific T cells will be necessary if we are to understand localized immunity, tolerance, and pathogenesis.

## Introduction

αβ T cells recognize antigens via the heterodimeric T cell receptor (TR), which is comprised of one alpha (TRA) chain and one beta (TRB) chain. The αβ TR is the most diverse protein in humans with the V(D)J recombination machinery producing 2.5 × 10^6^ unique TR structures from a theoretical catalog of 10^20^ structures [reviewed in Ref. (1)]. This hugely diverse base repertoire enables broad recognition of millions of self and foreign antigens.

The T cell repertoire can be defined by quantifying the expression of different T cell receptor beta variable (TRBV) proteins on the cell surface or from intracellular transcripts (1). Using this method, repertoire differences between blood and other anatomical sites can be investigated. For instance, large differences in T cell repertoire profile have been observed between peripheral blood mononuclear cells (PBMC) and colon tissue (2), with colon-resident T cells expressing a much more restricted TRBV signature (3). Compartment-specific T cell repertoires have also been noted at the fetal–maternal interface (4). This site is anatomically known as the decidua and only global TRBV usage in decidual mononuclear cells (DMC) have been examined to date. Multivariate dissection is important given that large clonal expansions are generally concentrated within the CD8+ (5) and regulatory T cell (Treg) (6) subsets.

Tregs play a defining role in pregnancy, overseeing maternal tolerance of the semi-allogeneic fetus (7). It has been shown that a significantly higher proportion of Tregs is present in the decidua compared with PBMC (8). In cases of pre-eclampsia and spontaneous abortion, this percentage is significantly reduced (9), emphasizing the critical role of Tregs in the maintenance of pregnancy.

Given that we know very little of the composition of T cell repertoires operating at decidua, we undertook immunoprofiling to define TR usage at the fetal–maternal interface within different T cell lineages. Using multiparametric flow cytometry-based phenotyping and TRBV dissection, we assessed the DMC repertoires of CD8+ CD4+ and Treg cells from women with healthy pregnancies and pre-eclampsia and compared these repertoires with corresponding subsets in peripheral blood.

## Materials and Methods

### Patients

Five term healthy pregnant women and three pre-eclamptic women in the third trimester of pregnancy from Nepean Hospital (Sydney, NSW, Australia) were included in this study. Pre-eclampsia was defined according to the International Society for the Study of Hypertension in Pregnancy criteria as onset of high blood pressure (>140/90) and proteinuria (>0.3 g/24 h) after 20 weeks of gestation (10). Patient details can be found in Table A2 in Appendix. Patients with diabetes, infectious conditions, chromosomal abnormalities, and/or morphological anomalies were excluded. This study was approved by the Human Research Ethics Committee of the Nepean Blue Mountains Local Health District, Penrith, NSW, Australia.

### Mononuclear cell isolation

DMC were obtained as previously described (11, 12). Briefly, decidua basalis was carefully dissected from the maternal surface of freshly delivered placenta. This was washed with PBS until the eluent was macroscopically clear of blood. The tissue was then minced by a pair of fine scissors to 1–2 mm pieces. A cocktail of collagenase (300 U/ml), hyaluronidase (2 mg/ml), DNase (50 μg/ml) (Sigma-Aldrich) added to RPMI-1640 containing 2 mM l-Glutamine, 100 U/ml penicillin, 100 μg/ml streptomycin, and 5% fetal calf serum was used for enzymatic digestion of decidual tissue. 10 ml of this mixture was used per 1 g of wet tissue weight, incubated at 37°C with gentle rotation. A regimen of 3 × 20 min pulsed digestion followed by 1 h digestion was performed. At the end of each period of digestion, supernatant containing released cells was removed. This was then washed once in PBS. At the end of the digestion, all washed supernatants were combined and filtered through a 70-μm sieve. Mononuclear cells were subsequently isolated using Ficoll–Hypaque (Amersham Pharmacia) density gradient centrifugation. DMC were then isolated using density gradient centrifugation over Ficoll–Hypaque. All samples were cryopreserved in RPMI-1640 containing 20% fetal bovine serum and 10% dimethyl sulfoxide (Sigma-Aldrich) and stored in liquid nitrogen.

### T cell repertoire immunoprofiling

Thawed PBMC or DMC were incubated for 30 min at 4°C with live/dead fixable aqua dead cell stain (Life Technologies), CD4 Alexa Fluor 700, CD8 Cy5.5-PerCP, and CD25 Cy7-phycoerythrin (BioLegend). Each sample was then divided and labeled for 30 min at 4°C with 1 of 25 phycoerythrin- or fluorescein isothiocyanate-labeled TRBV-specific mAb (Table A1 in Appendix). Nomenclature used in this study is based on IMGT^®^, the international ImMunoGeneTics information system^®^, http://www.imgt.org (13). Functional TRBV genes (14) not covered by this panel include; TRBV5-4, TRBV5-8, TRBV6-1, TRBV6-2, TRBV6-3, TRBV6-4, TRBV6-8, TRBV7-3, TRBV7-4, TRBV7-6, TRBV7-7, TRBV7-8, TRBV7-9, TRBV10-1, TRBV10-2, TRBV11-1, TRBV11-3, TRBV12-5, TRBV15, TRBV16, TRBV24-1, and TRBV29-1. After washing with PBS, the FoxP3 Fix/Perm Buffer Set and FoxP3 Alexa Fluor 647 mAb (BioLegend) were used to label intracellular FoxP3, according to the manufacturer’s protocol. Cells were analyzed on a FACSCanto II flow cytometer using FACSDiva software (BD Biosciences). Flow cytometry data were analyzed and presented using FlowJo software (TreeStar). A representative example of cell gating can be found in Data Sheet 2 in Supplementary Material.

## Results and Discussion

Five term healthy pregnant women and three pre-eclamptic women were enrolled in the study. Blood was drawn prior to delivery and PBMC isolated by density gradient centrifugation. DMC were obtained from freshly delivered placenta by tissue digestion and density gradient centrifugation as described in Section “Materials and Methods.” To compare the TRBV repertoires of different T cell subsets at each site, we employed mutiparametric flow cytometry and intracellular staining. Here, a panel of TRBV-specific mAb was combined with the phenotypic makers CD8, CD4, forkhead box P3 (FoxP3), and CD25 to dissect out TR repertoires of CD8+, CD4+, and Treg (CD4+ FoxP3+ CD25+) subsets. Although the antibody panel did not cover all TRBV gene products, TRBV profiling identified an average of 61% of the population for CD8+ T cells, 71% for CD4+ T cells, and 83% for Tregs (data not shown). The frequency of each TRBV subgroup as a percentage of the identified repertoire was calculated for each T cell subset. Frequencies were then compared between DMC and PBMC to determine the absolute difference in frequency for each TRBV subgroup within each T cell subset (Data Sheet 1 in Supplementary Material).

**Figure 1:**
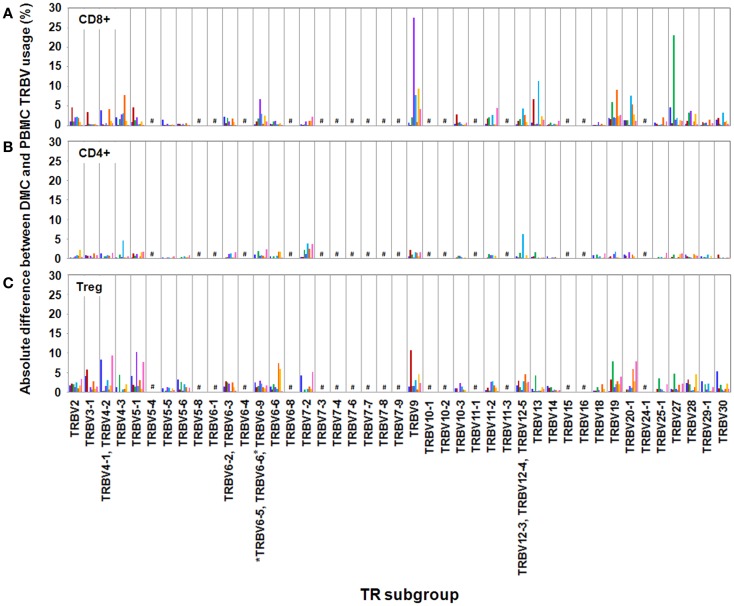
**Absolute differences between DMC and PBMC TRBV subgroup usage for each T cell subset**. For each patient, the percentage of the identified repertoire represented by each TRBV subgroup in DMC subsets was compared with that of T cell subsets within PBMC. These absolute differences in TRBV frequency between PBMC and DMC are shown for each donor, within **(A)** CD8+ T cells, **(B)** CD4+ T cells, and **(C)** Treg cells. Genes encoding TRBV for which no antibody was included or available are indicated by a #. TRBV gene names are according to the IMGT nomenclature (13, 14). *Although clone IMMU 222 has previously thought to recognize TRBV6-6, in addition to TRBV6-5 and TRBV6-9 (15), our data suggest that IMMU 222 is not TRBV6-6-specific, as dual staining was not observed using both IMMU 222 and JU74.3 mAbs.

We observed variation in TRBV usage between DMC and PBMC across all donors and across all T cell subsets (Figure 1). However, the degree of variation was clearly partitioned by T cell subset. CD4+ T cells showed the least disparity between anatomical sites with TRBV subgroups exhibiting <5% absolute variation in all cases but one. Interestingly, across both cohorts, the total CD4 compartment was surprisingly stable between the DMC and PBMC. Intriguingly, this repertoire stability included prominent TRBV5-1, TRBV19, and TRBV20 populations even between genetically diverse mothers (Data Sheet 1 in Supplementary Material).

In contrast with the CD4+ subset, CD8+ and Treg subsets exhibited considerable TR variation between anatomical sites. Between DMC and PBMC, CD8+ repertoire variation was striking with absolute differences in *single* TRBV subgroups up to 28% of the total compartment (Figure 1). For example, in donor H3, one in three CD8+ T cells in the DMC bore a receptor expressing TRBV27. This TRBV frequency at the decidua was more than threefold higher than peripheral blood (Data Sheet 1 in Supplementary Material). Interestingly, TRBV27 enrichment within DMC was also noted in donor H1. However, consistent TRBV27 enrichment was not seen across all donors precluding designation of this subgroup as an immunological fixture at the decidua in non-related humans. Other notable TRBV deviations between CD8+ compartments included a large TRBV9 expansion in the peripheral blood of donor H4. This population comprised 38% of the total CD8+ cells in PBMC, compared with 10% of total cells at the DMC (Data Sheet 1 in Supplementary Material). Further DMC-specific enrichment was observed for TRBV9 in donor H5 and pre-eclampsia donor P2 that was up to twofold higher than in peripheral blood (Data Sheet 1 in Supplementary Material).

Akin to the CD8+ subset, the Treg subset also exhibited considerable TR variation between DMC and PBMC with absolute differences of *single* TRBV subgroups up to 15% of the total compartment. Also of note, all donors except H5 exhibited a >5% absolute deviation in one or more TRBV subgroups between compartments. Notable DMC-specific enrichments included a dominant TRBV9 expansion in donor H2 that was 3.5-fold higher than in peripheral blood (Data Sheet 1 in Supplementary Material). Additionally, the TRBV4 subgroup represented by the identification of TRBV4-1, TRBV4-2, TRBV4-3 was clearly enriched in the DMC of donor H1 and TRBV5-1 was enriched in the DMC of both donor H4 and pre-eclampsia donor P3 (Data Sheet 1 in Supplementary Material). One curious observation in the Treg subset was the dominance of the TRBV20-1 gene across all examined donors (Data Sheet 1 in Supplementary Material). In both PBMC and DMC compartments, TRBV20-1 positive T cells were extremely common representing 11–24% of the total Treg repertoire. Interestingly, we recently observed this TRBV20-1 immunodominance in Treg using an alternate immunoprofiling method of high throughput TR sequencing (16). Here, TRBV20-1 was used extensively by Tregs [29.5% of the sequences assigned to clonotypes defined as IMGT clonotypes (AA) (16)], but less by the CD8+ and CD4+ repertoires (20 and 16%, respectively). Across all subsets, TRBV20-1 represented 21.9% of all functional clonotypes. Additionally, another high throughput TR sequencing study using pooled PBMC from 500 individuals revealed that TRBV20-1 was commonly used (average 24.6% of total TRBV genes) (17). These common observations tentatively suggest that there is something fundamental about the TRBV20-1 gene in T cell and Treg ontogeny and function.

**Figure 2:**
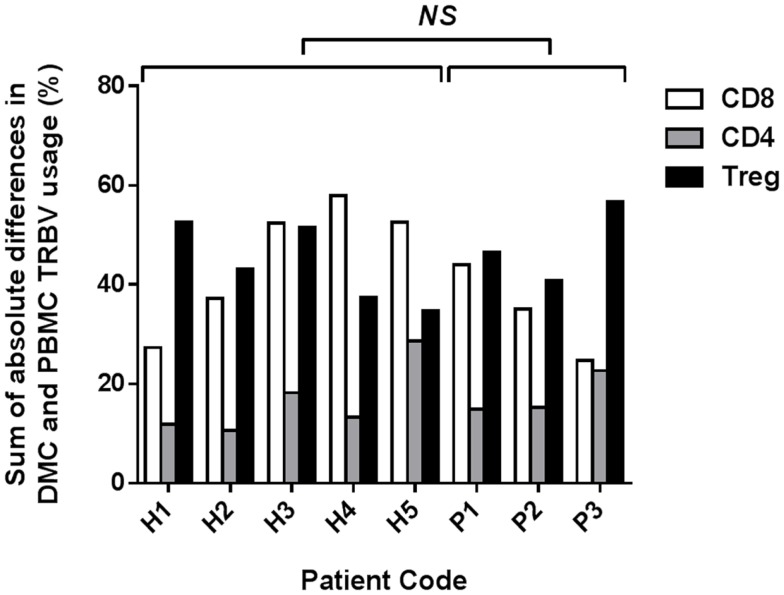
**Sum of absolute differences between TRBV usage of CD8+, CD4+, and Treg subsets in DMC and PBMC**. For each patient, the percentage of the identified repertoire represented by each TRBV subgroup in DMC subsets was compared with that of T cell subsets within PBMC. The sum of the absolute differences in TRBV frequency for all 25 subgroups analyzed was calculated, for each T cell subset, to determine the total difference between repertoires of T cells from DMC and PBMC. No significant difference (NS) was found between healthy and pre-eclamptic patients, for any T cell subset (Student’s*t*-test).

To determine the overall disparity between TRBV repertoires of PBMC and DMC from each patient, the sum of the absolute differences in frequency for each TRBV subgroup was calculated (Figure 2). The mean sum of absolute differences was 17% for CD4+ T cells (range 11–29%), 41% for CD8+ T cells (range 25–58%), and 45% for Tregs (range 35–57%). The sum of absolute differences was compared between healthy pregnant donors and patients with pre-eclampsia, and no significant differences were identified for the three T cell subsets assessed (Student’s *t*-test). While no difference in global repertoire was observed between healthy pregnancy and pre-eclampsia, it will be important for future studies to determine clonotypic usage of these subsets during health and disease.

The idea that TRBV repertoire is partitioned by anatomical site was proposed by Ochsenreither et al., who compared T cells from healthy colon tissue and colorectal carcinoma with PBMC from the same donor (3). They found that the TR repertoire differed between sites, with the mucosal cells being more limited in TRBV diversity. The authors proposed that colon-specific antigens were responsible for shaping the local T cell repertoire. Our data similarly suggest that exposure of T cells to unique antigens in the decidua could be underlying the variation seen between T cell repertoires in these two compartments.

Within the decidual Treg population, it is likely that expanded TRBV subgroups are critical for maternal tolerance of the fetus. Identifying the antigen specificity of cells within these subgroups would help clarify their functional role within the decidua. Given the prominent differences between T cell subset repertoires of the PBMC and DMC, further investigation is warranted. Comprehensive clonotypic characterization across T cell subsets is the logical next step, ideally using unbiased high throughput TR sequencing technology (16). Studies such as these will help categorize the newly classified tissue resident T cell subsets (18) and may assist in understanding the TRBV20 enrichment in Treg. They may also identify subsets of “innate like” αβ T cells that are known to engage an emerging suite of non-classical major histocompatibility (MH) proteins (19).

Although healthy donors and patients with pre-eclampsia exhibited the same level of variation in TRBV repertoire between the two compartments, this study does not rule out that the decidual Treg population in pre-eclamptic patients lacks essential clonotypes required for maternal tolerance. Previous studies have shown that number (8) and phenotypic composition (20) of decidual Tregs differ between healthy and pre-eclamptic pregnancies, but clonotypic diversity is yet to be comprehensively assessed. These studies will be important since it is known that the “type” of clonotype chosen by the host can determine clinical outcome across many disease states (1).

In summary, we have shown that T cell repertoires within decidual tissue differ substantially from those in peripheral blood, particularly in the CD8+ and Treg subsets. Further study of expanded TRBV subgroups within decidual tissue, and Tregs in healthy pregnant donors and pre-eclampsia patients could provide critical insights into the mechanisms of maternal tolerance and the etiology of pre-eclampsia.

## Author Contributions

Brigitte Santner-Nanan, Peter Hsu, Steven Joung, and Ralph Nanan acquired and processed patient samples. Michelle A. Neller and John J. Miles acquired, analyzed, and interpreted data. Scott R. Burrows, John J. Miles, Brigitte Santner-Nanan, Rebekah M. Brennan, and Ralph Nanan contributed to project design and data interpretation. Michelle A. Neller, Scott R. Burrows, and John J. Miles wrote the manuscript.

## Conflict of Interest Statement

The authors declare that the research was conducted in the absence of any commercial or financial relationships that could be construed as a potential conflict of interest.

## Supplementary Material

The Supplementary Material for this article can be found online at http://www.frontiersin.org/Journal/10.3389/fimmu.2014.00033/abstract

## Appendix

**Table A1:** **Monoclonal antibody characteristics**.

Reagent antibody	TRBV gene specificity^d^	Clone name	Fluorochrome	Catalog number^a^
Vbeta 1	TRBV9	BL37.2	FITC	MCA1591F^c^
Vbeta 2	TRBV20-1	MPB2D5	PE	IM2213
Vbeta 3	TRBV28	CH92	FITC	IM2372
Vbeta 4	TRBV29-1	WJF24	PE	IM3602
Vbeta 5.1	TRBV5-1	IMMU 157	PE	IM2285
Vbeta 5.2	TRBV5-6	36213	PE	IM2286
Vbeta 5.3	TRBV5-5	3D11	PE	IM2002
Vbeta 6.7	TRBV7-2	OT145	FITC	TCR2657^b^
Vbeta 7.1	TRBV4-1, TRBV4-2, TRBV4-3	ZOE	PE	IM2287
Vbeta 7.2	TRBV4-3	ZIZOU4	PE	IM3604
Vbeta 8	TRBV12-3, TRBV12-4	56C5.2	PE	IM2289
Vbeta 9	TRBV3-1	FIN9	PE	IM2003
Vbeta 11	TRBV25-1	C21	PE	IM2290
Vbeta 12	TRBV10-3	VER2.32.1	PE	IM2291
Vbeta 13.1	TRBV6-5, TRBV6-6^e^, TRBV6-9	IMMU 222	PE	IM2292
Vbeta 13.2	TRBV6-2, TRBV6-3	H132	PE	IM3603
Vbeta 13.6	TRBV6-6	JU74.3	FITC	IM1330
Vbeta 14	TRBV27	CAS1.1.3	PE	IM2047
Vbeta 16	TRBV14	TAMAYA1.2	FITC	IM1560
Vbeta 17	TRBV19	E17.5F3.15.13	PE	IM2048
Vbeta 18	TRBV18	BA62.6	PE	IM2049
Vbeta 20	TRBV30	ELL1.4	PE	IM2295
Vbeta 21.3	TRBV11-2	IG125	FITC	IM1483
Vbeta 22	TRBV2	IMMU 546	PE	IM2051
Vbeta 23	TRBV13	AF23	PE	IM2004

*^a^TRBV mAb sourced from Beckman Coulter unless specified*.

*^b^Pierce endogen*.

*^c^Serotec*.

*^d^TRBV gene specificity is from IMGT^®^, http://www.imgt.org, IMGT repertoire (IG and TR) > 6. Gene regulation and expression > reagents monoclonal antibodies’ (13, 14), except for IM3602 and IM3604 (not published)*.

*^e^IMMU 222 may not be TRBV6-6-specific (see Data Sheet 1 in Supplementary Material)*.

**Table A2:** **Patient characteristics**.

	Healthy pregnancy	Pre-eclampsia
Sample numbers	5	3
Age (years)	28.0 (±2.7)	28.3 (±5.2)
Gestational age at delivery (weeks)	38.8 (±0.35)	36.9 (±1.5)
Birth weight (grams)	3480 (±354)	2996 (±512)

*Numbers shown represent means (±standard deviations)*.

## References

[1] Miles JJ Douek DC Price DA Bias in the alphabeta T-cell repertoire: implications for disease pathogenesis and vaccination. Immunol Cell Biol (2011) 89(3):375–87 10.1038/icb.2010.139 21301479

[2] Bennet JD Brown WR Kotzin BL Regional variation in the lamina propria T cell receptor V beta repertoire in normal human colon. Clin Immunol (1999) 90(1):38–46 10.1006/clim.1998.4627 9884351

[3] Ochsenreither S Fusi A Wojtke S Busse A Nussler NC Thiel E Comparison of T-cell receptor repertoire restriction in blood and tumor tissue of colorectal cancer patients. J Transl Med (2010) 8:35 10.1186/1479-5876-8-35 20385014 PMC2873372

[4] Dokouhaki P Moghaddam R Rezvany M Ghassemi J Novin MG Zarnani A Repertoire and clonality of T-cell receptor beta variable genes expressed in endometrium and blood T cells of patients with recurrent spontaneous abortion. Am J Reprod Immunol (2008) 60(2):160–71 10.1111/j.1600-0897.2008.00608.x 18705843

[5] Neller MA Burrows JM Rist MJ Miles JJ Burrows SR High frequency of herpesvirus-specific clonotypes in the human T cell repertoire can remain stable over decades with minimal turnover. J Virol (2013) 87(1):697–700 10.1128/JVI.02180-12 23077319 PMC3536364

[6] Relland LM Williams JB Relland GN Haribhai D Ziegelbauer J Yassai M The TCR repertoires of regulatory and conventional T cells specific for the same foreign antigen are distinct. J Immunol (2012) 189(7):3566–74 10.4049/jimmunol.1102646 22933635 PMC3538134

[7] Aluvihare VR Kallikourdis M Betz AG Regulatory T cells mediate maternal tolerance to the fetus. Nat Immunol (2004) 5(3):266–71 10.1038/ni1037 14758358

[8] Tilburgs T Roelen DL van der Mast BJ van Schip JJ Kleijburg C de Groot-Swings GM Differential distribution of CD4(+)CD25(bright) and CD8(+)CD28(-) T-cells in decidua and maternal blood during human pregnancy. Placenta (2006) 27(Suppl A):S47–53 10.1016/j.placenta.2005.11.008 16442616

[9] Santner-Nanan B Peek MJ Khanam R Richarts L Zhu E Fazekas de St Groth B Systemic increase in the ratio between Foxp3+ and IL-17-producing CD4+ T cells in healthy pregnancy but not in preeclampsia. J Immunol (2009) 183(11):7023–30 10.4049/jimmunol.0901154 19915051

[10] Report of the National High Blood Pressure Education Program Working Group on High Blood Pressure in Pregnancy. Am J Obstet Gynecol (2000) 183(1):S1–22 10920346

[11] Vince GS Starkey PM Jackson MC Sargent IL Redman CW Flow cytometric characterisation of cell populations in human pregnancy decidua and isolation of decidual macrophages. J Immunol Methods (1990) 132(2):181–9 10.1016/0022-1759(90)90028-T 2145368

[12] Hsu P Santner-Nanan B Dahlstrom JE Fadia M Chandra A Peek M Altered decidual DC-SIGN+ antigen-presenting cells and impaired regulatory T-cell induction in preeclampsia. Am J Pathol (2012) 181(6):2149–60 10.1016/j.ajpath.2012.08.032 23063509

[13] Lefranc MP Giudicelli V Ginestoux C Jabado-Michaloud J Folch G Bellahcene F IMGT, the international ImMunoGeneTics information system. Nucleic Acids Res (2009) 37:D1006–12 10.1093/nar/gkn838 18978023 PMC2686541

[14] Lefranc MP Lefranc G The T Cell Receptor Facts Book. London: Academic Press (2001).

[15] Ferradini L Roman-Roman S Azocar J Michalaki H Triebel F Hercend T Studies on the human T cell receptor alpha/beta variable region genes. II. Identification of four additional V beta subfamilies. Eur J Immunol (1991) 21(4):935–42 10.1002/eji.1830210412 1826889

[16] Li S Lefranc MP Miles JJ Alamyar E Giudicelli V Duroux P IMGT/HighV QUEST paradigm for T cell receptor IMGT clonotype diversity and next generation repertoire immunoprofiling. Nat Commun (2333) 2013:4 10.1038/ncomms3333 23995877 PMC3778833

[17] Freeman JD Warren RL Webb JR Nelson BH Holt RA Profiling the T-cell receptor beta-chain repertoire by massively parallel sequencing. Genome Res (2009) 19(10):1817–24 10.1101/gr.092924.109 19541912 PMC2765271

[18] Mueller SN Gebhardt T Carbone FR Heath WR Memory T cell subsets, migration patterns, and tissue residence. Annu Rev Immunol (2013) 31:137–61 10.1146/annurev-immunol-032712-095954 23215646

[19] Adams EJ Luoma AM The adaptable major histocompatibility complex (MHC) fold: structure and function of nonclassical and MHC class I-like molecules. Annu Rev Immunol (2013) 31:529–61 10.1146/annurev-immunol-032712-095912 23298204

[20] Steinborn A Schmitt E Kisielewicz A Rechenberg S Seissler N Mahnke K Pregnancy-associated diseases are characterized by the composition of the systemic regulatory T cell (Treg) pool with distinct subsets of Tregs. Clin Exp Immunol (2012) 167(1):84–98 10.1111/j.1365-2249.2011.04493.x 22132888 PMC3248090

