# Supplementary material for: Multivariate Analysis Using High Definition Flow Cytometry Reveals Distinct T Cell Repertoires between the Fetal–Maternal Interface and the Peripheral Blood
Source: Front Immunol. 2014 Feb 5;5:33. doi: 10.3389/fimmu.2014.00033 (PMC3913911; doi:10.3389/fimmu.2014.00033)
Supplement: Supplementary file 1 [file 76688_Miles_DataSheet1.PDF]

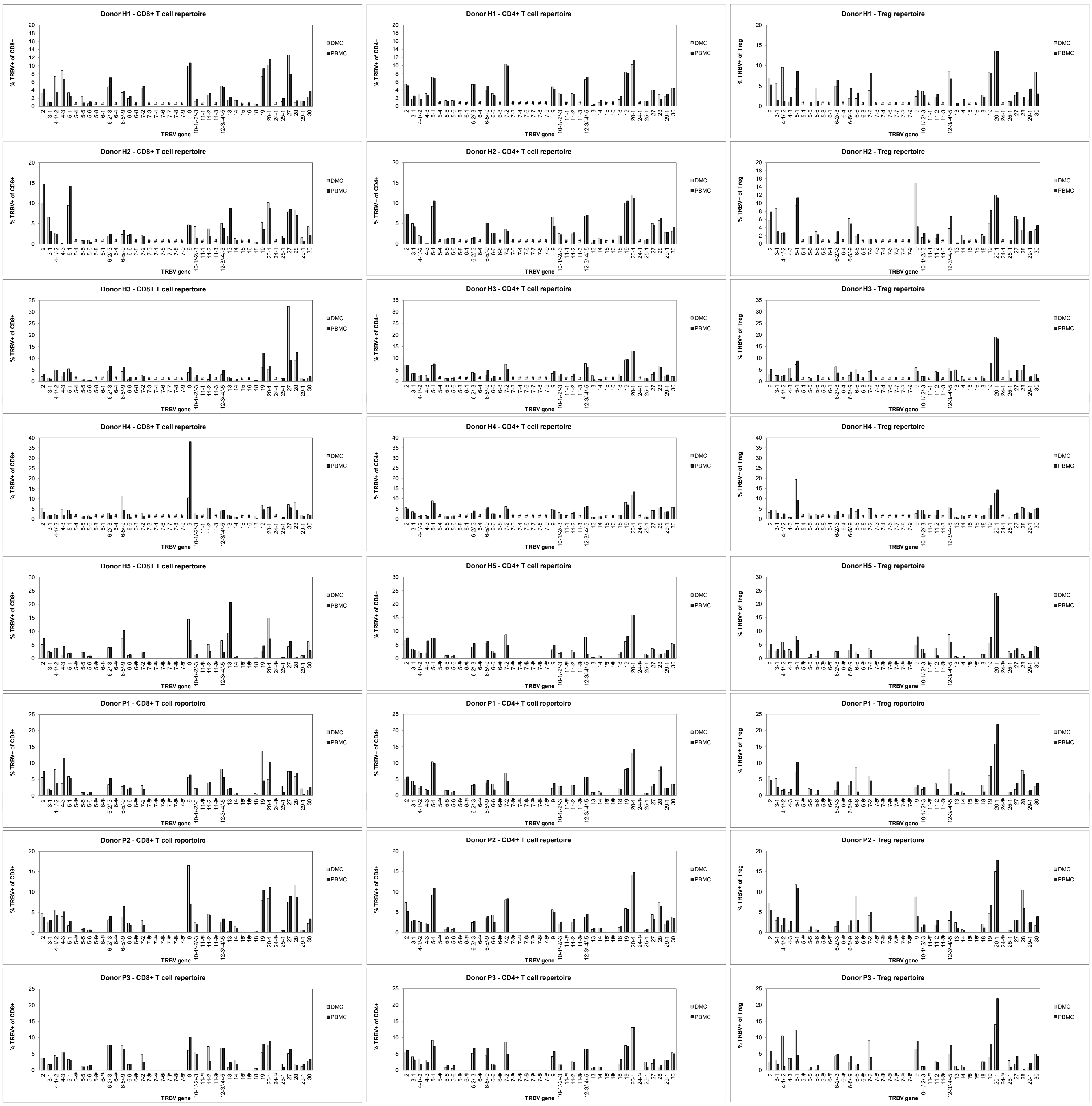

Data Set 1. Frequency of each TRBV family within T cell subsets. PBMC and DMC from each donor were labelled with mAb specific for phenotypic markers, in addition to a panel of 25 TRBV mAb, then analysed on a FACSCanto II flow cytometer. The frequency of each TRBV family as a percentage of the identified repertoire for CD8+, CD4+ and Treg cells is shown for PBMC and DMC subsets from each patient. Where an antibody recognises multiple TRBV gene products, the gene numbers are listed in a single column. Genes encoding TRBVs for which no antibody was included or available are indicated by a #.
