# Supplementary figures and images for: Multivariate Analysis Using High Definition Flow Cytometry Reveals Distinct T Cell Repertoires between the Fetal–Maternal Interface and the Peripheral Blood
Source: Front Immunol. 2014 Feb 5;5:33. doi: 10.3389/fimmu.2014.00033 (PMC3913911; doi:10.3389/fimmu.2014.00033)

# Data Set 2

**A**

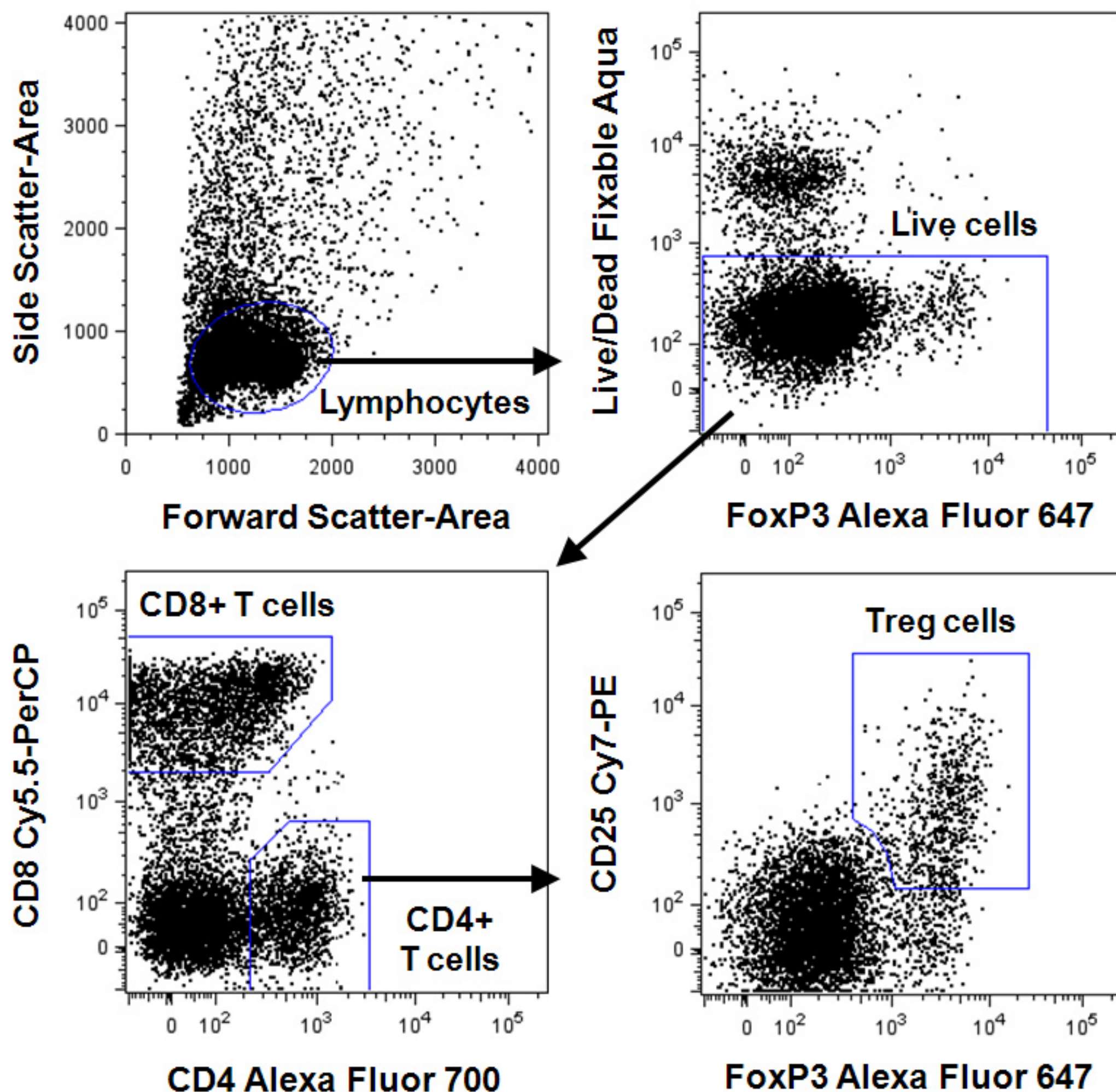

**B**

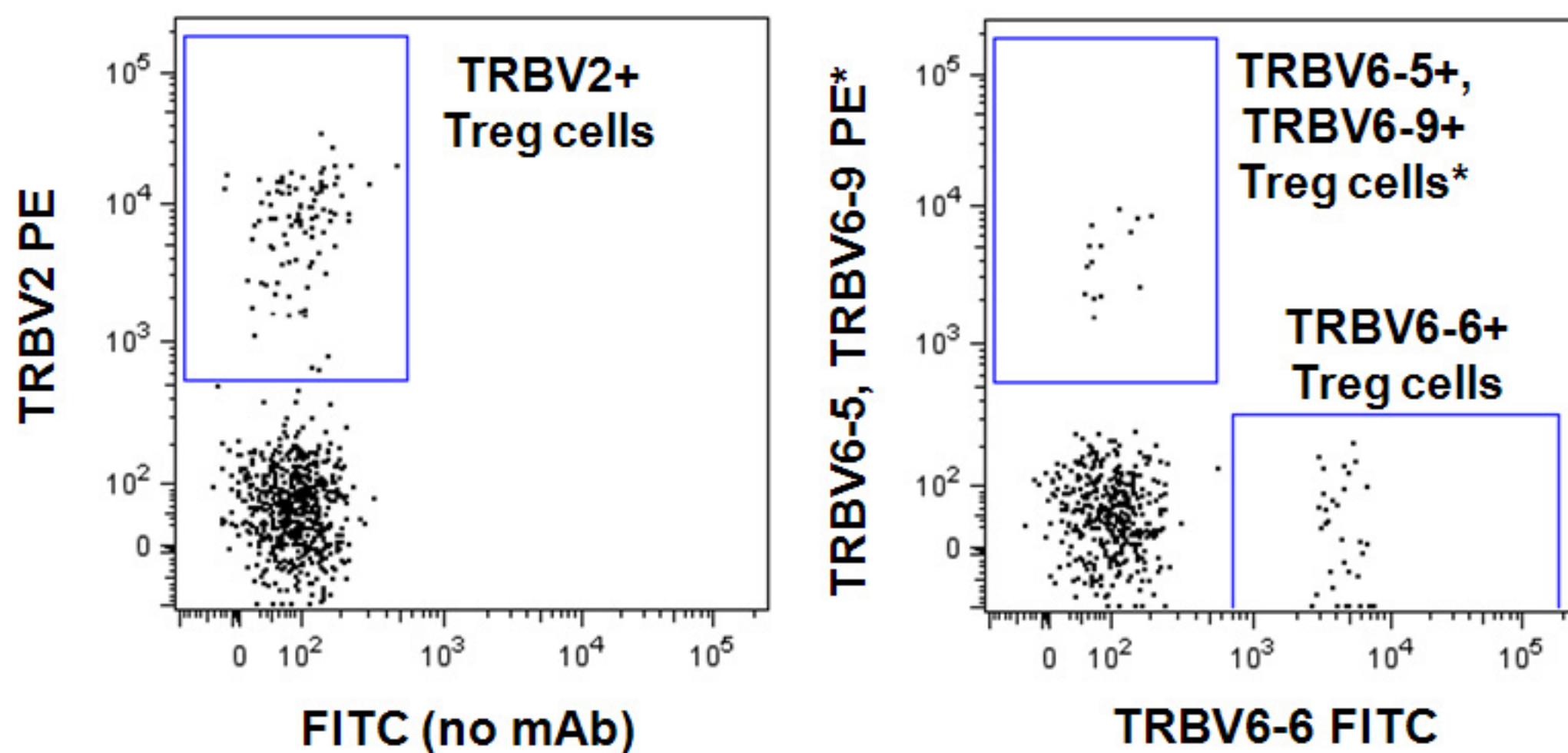

Supplement: Supplementary file 2 [file 76688_Miles_DataSheet2.PDF]
